# Nanostructured Lipid Carrier Gel for the Dermal Application of Lidocaine: Comparison of Skin Penetration Testing Methods

**DOI:** 10.3390/pharmaceutics11070310

**Published:** 2019-07-02

**Authors:** Stella Zsikó, Kendra Cutcher, Anita Kovács, Mária Budai-Szűcs, Attila Gácsi, Gabriella Baki, Erzsébet Csányi, Szilvia Berkó

**Affiliations:** 1 Institute of Pharmaceutical Technology and Regulatory Affairs, Faculty of Pharmacy, University of Szeged, 6720 Szeged, Hungary; 2 Frederic and Mary Wolfe Center, College of Pharmacy and Pharmaceutical Sciences, University of Toledo, Toledo, OH 43614, USA

**Keywords:** dermal drug delivery, diffusion cell, Franz diffusion, Skin-PAMPA, Strat-M^®^ membrane, nanocarrier

## Abstract

The aim of this research was to investigate the stability of a lidocaine-loaded nanostructured lipid carrier dispersion at different temperatures, formulate a nanostructured lipid carrier gel, and test the penetration profile of lidocaine from the nanostructured lipid carrier gel using different skin penetration modeling methods. The formulations were characterized by laser diffraction, rheological measurements and microscopic examinations. Various in vitro methods were used to study drug release, diffusion and penetration. Two types of vertical Franz diffusion cells with three different membranes, including cellulose, Strat-M^®^, and heat separated human epidermis were used and compared to the Skin-parallel artificial membrane permeability assay (PAMPA) method. Results indicated that the nanostructured lipid carrier dispersion had to be gelified as soon as possible for proper stability. Both the Skin-PAMPA model and Strat-M^®^ membranes correlated favorably with heat separated human epidermis in this research, with the Strat-M^®^ membranes sharing the most similar drug permeability profile to an ex vivo human skin model. Our experimental findings suggest that even when the best available in vitro experiment is selected for modeling human skin penetration to study nanostructured lipid carrier gel systems, relevant in vitro/in vivo correlation should be made to calculate the drug release/permeation in vivo. Future investigations in this field are still needed to demonstrate the influence of membranes and equipment from other classes on other drug candidates.

## 1. Introduction

Topical and transdermal formulations are widely used for delivering drugs to the skin and underlying tissue, or through the skin for systemic action. The vast majority of topically applied formulations are semisolids, including creams, ointments and gels, which offer suitable residence time on the skin, and are usually well accepted by the patients. Topical formulations deliver the active pharmaceutical ingredients (APIs) into different layers of the skin, thus enabling several diseases to be prevented and/or cured. The beginning, duration and strength of the therapeutic effect depend on the efficacy of three consecutive processes: (1) The release of the API from the carrier system; (2) penetration/diffusion of the API into the stratum corneum or other skin layers; (3) exertion of a pharmacological effect at the target point. All these effects determine the safety-efficacy profile of a product [1,2,3]. For most skin problems or diseases, the target point is the stratum corneum, viable epidermis or dermis.

Modeling of penetration through the skin is discussed by numerous directives [4,5,6,7,8]. The in vitro method for skin penetration modeling is included in OECD Guideline 428 [5]. Advantages of the in vitro method are that measurements can be carried out on human skin samples among other potential membranes; multiple tests can be performed on a skin sample from the same donor; several formulations can be tested at the same time; there is no need for radio-labelling the test material; and there are no ethical issues.

Topical semisolid products are complex formulations with a complex structure. Physical properties of the composition depend on several factors, including particle size of dispersed particles, surface tension between the phases, fractional distribution of the drug between the phases, and rheological behavior of the product. These properties collectively determine the in vitro dissolution profile, together with other characteristics. The amount of API released in vitro is an important quality characteristic of a product. Modeling of penetration through the skin is a complex challenge. The device and the membrane, along with the properties of the product influence how the system can be tested most effectively. There are various types of equipment with which the diffusion and penetration of drug carrier systems can be studied. Human skin tests give the most relevant information; however, because of the high cost, it is a generally accepted approach to choose simpler methods in the early stages of formulation development. Previously, several simple, reliable, reproducible and validated methods were described for drug liberation by a vertical diffusion cell (VDC) using a synthetic membrane [9,10,11]. In vitro drug release testing is a widely used and reliable tool for evaluating products.

The use of Franz diffusion cells to evaluate skin permeability has developed into a major research methodology. With Franz diffusion studies, diffusion and penetration of an API through the skin and the relationships among the skin, drug and formulation can be investigated [12,13,14,15,16,17,18,19]. Franz diffusion cells are available with multiple types of automatic sampler systems. A lot of different membranes, including artificial and biological membranes can be applied on the Franz cells. Artificial membranes provide a simple and reproducible alternative way to study the basic physicochemical mechanisms of the drug diffusion, including interactions between the carrier and the membrane. They are suitable for early-stage drug development studies [13]. The Strat-M^®^ membrane, built by two polyether sulfone layers, was specifically designed for skin penetration studies. The layers are impregnated with a mixture of synthetic lipids, and a polyolefin layer is located underneath. Moving downwards into the membrane layers, the size of the pores increases [20]. Transdermal diffusion and safety of APIs, excipients, functional cosmetic ingredients, cosmetic active substances, and finished products can be tested using the Strat-M^®^ membrane. Advantages include that its diffusion properties are highly correlated with human diffusion studies, and it is characterized by minimal variability. It is also long-lasting and requires no special storage conditions or preparation for its use [20,21,22,23,24]. In addition to synthetic membranes, human skin membranes can also be used to model skin penetration. In the development of dermal preparations, it is not sufficient to perform membrane diffusion studies. It is necessary to do permeation studies through the biological membrane since the interaction of the API and carrier system with the skin can also be investigated, and it can be determined whether and to what extent the skin acts as a reservoir for the API. Usually, skin sections are obtained from plastic surgery, and a heat-separated epidermis is used as a membrane [13,25,26].

The parallel artificial membrane permeability assay (PAMPA) is a device that is adapted to simulate penetration of a product into the skin. For Skin-PAMPA, the bottom part of the PAMPA sandwich is substituted by a special donor plate, which was designed to study semi-solid products. It contains synthetic ceramides and has similar penetration properties to full-thickness skin. Synthetic lipids are easier to store, and their stability is more favorable [27,28,29,30]. The biomimetic model is able to determine in vitro human skin data based on the tests and can be a good alternative to skin permeability testing [17,28,31].

Lidocaine is a commonly used numbing agent before local interventions. Delivering lidocaine to the skin from a specific vehicle provides a numbing/pain relief effect to that area. It is possible to localize the numbing effect onto a smaller skin area without reaching the muscle. By concentrating the numbing effect to the skin, the patient is able to retain full motor function of the area to which lidocaine is applied.

Our research group previously formulated and compared different drug carrier systems for the dermal delivery of lidocaine, including a conventional hydrogel, an oleogel, a lyotropic liquid crystalline gel and nanostructured lipid carrier (NLC) gel. It was concluded that the penetration of lidocaine from the NLC gel was the most significant, and the skin hydrating and occlusive effect made it a favorable carrier system [25,32]. In addition, several articles in the literature featured lidocaine-filled NLCs or NLC gels [33,34,35,36,37]. Therefore, a lidocaine-loaded NLC and NLC gel was selected for the current investigations.

In this study, our aims were to investigate the stability of a lidocaine-loaded NLC dispersion at various storage temperatures, formulate an NLC gel, and evaluate the penetration profile of lidocaine from the NLC gel. Two types of vertical Franz diffusion cells with three different membranes, including a cellulose membrane, Strat-M^®^ membrane, and heat separated human epidermis were used to study the drug release and permeation. Additionally, the NLC gels were evaluated using the Skin-PAMPA method, and the three methods were compared.

## 2. Materials and Methods

### 2.1. Materials

Lidocaine base and glycerol were obtained from Hungaropharma Ltd. (Budapest, Hungary). Miglyol^®^ 812 N (caprylic/capric triglyceride) was a gift from Sasol GmbH (Hamburg, Germany). Cremophor^®^ RH 60 (PEG-60 Hydrogenated Castor Oil; HLB value: 15–17) was kindly supplied by BASF SE Chemtrade GmbH (Ludwigshafen, Germany). Apifil^®^ (PEG-8 Beeswax) was a gift from Gattefossé (St. Priest, France). Methocel™ E4M (hydroxypropyl methylcellulose) was from Colorcon (Budapest, Hungary). The water used was purified and deionized with the Milli-Q system from Millipore (Milford, MA, USA). The cellulose acetate filter (Porafil membrane filter, cellulose acetate, pore diameter: 0.45 μm) was obtained from Macherey-Nagel GmbH & Co. KG (Düren, Germany). The Strat-M^®^ membrane (Strat-M^®^ Membrane, Transdermal Diffusion Test Model, 25 mm) was from Merck KGaA (Darmstadt, Germany). Excised human skin was obtained from a Caucasian female patient by routine plastic surgery procedure in the Department of Dermatology and Allergology, University of Szeged. The ex vivo skin penetration test does not need ethical permission, and patient’s consent according to the Act CLIV of 1997 on health, Section 210/A in Hungary. The local ethical committee (Ethical Committee of the University of Szeged, Albert Szent-Györgyi Clinical Center) was informed about the ex vivo skin penetration studies (Human Investigation Review Board license number: 83/2008). The Skin-PAMPA sandwiches (P/N: 120657), hydration solution (P/N: 120706) and stirring bars (P/N: 110066) were purchased from Pion, Inc (Woburn, MA, USA). The UV plates (UV-star micro plate, clear, flat bottom, half area) were from Greiner Bio-one (Kremsmünster, Austria).

### 2.2. Methods

#### 2.2.1. Preparation of the NLC

NLCs are colloidal carriers which were introduced in the early 1990s. They are derived from o/w emulsions by replacing the liquid lipid with a solid lipid at room temperature. The lipophlic phase of NLC included Apifil, Cremophor RH 60 and Miglyol 812 N, which were melted at 60 °C under controlled stirring. Then lidocaine was added to the melted lipid phase under similar conditions. Then purified water was added to the lipid phase to form the pre-emulsion. The pre-emulsion was ultrasonicated using a Hielscher UP200S compact ultrasonic homogenizer (Hielscher Ultrasonics GmbH, Teltow, Germany) for 10 min at 70% amplitude. At the end, the sample was cooled in ice to obtain the solid lipid particles (LID-NLC) [38]. For dermal application, a gel was formed at room temperature with glycerol and Methocel E4M. In the last step, the NLC dispersion was added to the gel (LID-NLC gel). A blank-NLC and blank-NLC gel were also prepared using the same procedure, but without adding lidocaine. Table 1 summarizes the formulations. The NLC formulations were stored at different temperatures 7 °C (cold) and 25 °C (room temperature).

#### 2.2.2. Laser Diffraction

Laser diffraction analysis was performed with a Malvern Mastersizer 2000 particle size analyzer provided with the Hydro SM wet dispersion unit (Malvern Instruments, Worcestershire, UK) to determine the particle size and stability of the NLC formulation at different temperatures (cold and room temperature). A few drops of the sample were placed inside the Hydro SM loaded with purified water, and the speed of the stirrer was set to 2000 rpm. Three values, namely d(0.1), d(0.5), and d(0.9), were evaluated, indicating that 10%, 50%, and 90% of the analyzed particles were below a certain size (diameter). The span value describing the width of the particle size distribution curve ((d(0.9) − d(0.1))/d(0.5)) was calculated as well.

#### 2.2.3. Rheological Measurements

Rheological measurements were performed with a Physica MCR101 rheometer (Anton Paar GmbH, Graz, Austria), employing a parallel plate geometry PP25 with a measuring gap of 0.1 mm to verify the proper consistency of the LID-NLC gel system for topical application. The blank-NLC gel was studied as well. Flow curves, viscosity curves and frequency sweep tests were obtained. The shear rate was increased from 0.1 to 100 1/s (up-curve) and then decreased from 100 to 0.1 1/s (down-curve) in CR mode. The shearing time in was 300 s. The measurements were carried out at 25 °C [39].

#### 2.2.4. Microscopic Examinations

The structure of the NLC samples was examined with a microscope (LEICA DM6 B, Leica Microsystems GmbH, Wetzlar, Germany) at room temperature. Magnifications were 100× and 200×.

#### 2.2.5. pH Measurements

For pH measurement, 10 g of each sample (NLC dispersion and NLC gel) was placed in a beaker, and the surface pH was measured using a Testo 206 pH meter (Testo SE & Co. KGaA, Lenzkirch, Germany) with a pH2 probe. Three parallel measurements were carried out at room temperature.

#### 2.2.6. Drug Diffusion and Penetration Studies

Three different methods were used to model and compare drug release and diffusion through the membrane and penetration into the skin from the LID-NLC gel (Table 2). The methods included two types of vertical Franz diffusion cells, namely Hanson Microette TM Topical & Transdermal Diffusion Cell System, and Logan Automated Dry heat sampling system. The third method used was the Skin-PAMPA method. On the Franz cells, the donor and acceptor phases were separated by either a synthetic cellulose acetate membrane, a Strat-M^®^ membrane or heat-separated human epidermis (HSE). A 0.3 g portion of sample was placed in the donor chamber on the membrane. The human skin was placed in a water bath (60 ± 0.5 °C), and the epidermis was separated from the dermis. Thermostated phosphate buffer solution (PBS pH 7.4 ± 0.15), made in-house, kept at 32 ± 0.5 °C was used as the acceptor phase. Membrane diffusion and skin penetration experiments lasted 24 h (sampling times: 0.5; 1; 2; 3; 4; 5; 6; 8; 10; 12; 16; 20; and 24 h). The stirring speed was 400 rpm. The concentration of the drug was measured spectrophotometrically with a Thermo Scientific Evolution 201 spectrometer with Thermo Insight v1.4.40 software package (Thermo Fisher Scientific, Waltham, MA, USA) at a wavelength of 262 nm (analytical parameters: Slope: 0.001738 ± 0.000016; SE: 4.554 × 10^−5^; SD: 0.0001205; LOD: 0.2339 µg/mL; LOQ: 0.7088 µg/mL). The Skin-PAMPA sandwiches were used after a 24-hour hydration period (Hydration Solution, Pion, Inc., Billerica, MA, USA). 70 µL of the NLC gels were applied to the well of the formulation plate as the donor phase. Phosphate buffer solution (PBS pH 7.4 ± 0.15), made in-house, was used as the acceptor phase. The top plate was filled with 250 µL of fresh acceptor solution, and a stirring bar was also used in each well. The Gut-Box™ from Pion, Inc. was used for stirring; the resultant sandwich was incubated at 32 °C for six hours. The Skin-PAMPA membrane is not designed for 24-h measurements, the membrane can provide relevant values for a shorter time. Therefore, the acceptor solution was examined after 0.5, 1, 2, 3, 4, 5 and 6 h of incubation. The quantity of the API was determined by UV spectroscopy at 262 nm using a Synergy HT UV plate reader by KC4 (BIO-TEK Instruments, Inc., Winooski, VT, USA) software. The quantity of the permeated API was expressed as µg/cm^2^ units.

#### 2.2.7. Statistical Analysis

Two-way ANOVA analysis of variance (Bonferroni post-test), working with Prism for Windows software (GraphPad Software Inc., La Jolla, CA, USA), was used to analyze the statistical difference between the samples. Results are shown as average of six parallel experiments and standard deviation (SD). Variations were considered significant if *p* < 0.05 *, *p* < 0.01 ** and *p* < 0.001 *** versus the control.

## 3. Results and Discussion

### 3.1. Particle Size Analysis

Usually light scattering methods, such as photon correlation spectroscopy and/or laser diffraction (LD) are used for particle size determination of lipid nanoparticles. LD is a powerful method that has a wider detection spectrum (20 nm–2.000 μm), and it is considered a better option for lipid nanoparticles in the upper nanometer and micrometer size ranges.

All NLC dispersions were in the nanosize range, between 97 and 163 nm (d(0.5)). The particle size of LID-NLC stored at room temperature was the smallest (97 nm), and it was stable for three days (the values of d(0.5) and d(0.9) were below 200 nm). On the fourth day, the d(0.9) values showed a significant increase (41.749 μm). The particle size of the NLC dispersion stored in the refrigerator increased after one day. The reason for this instability was that lidocaine crystallized in the refrigerator, and the formulation was not stable under these conditions. The exact parameters are summarized in Table 3. According to the particle size analysis, the holding time of the preparation was three days at room temperature.

### 3.2. Microscopic Study

**Figure 1:**
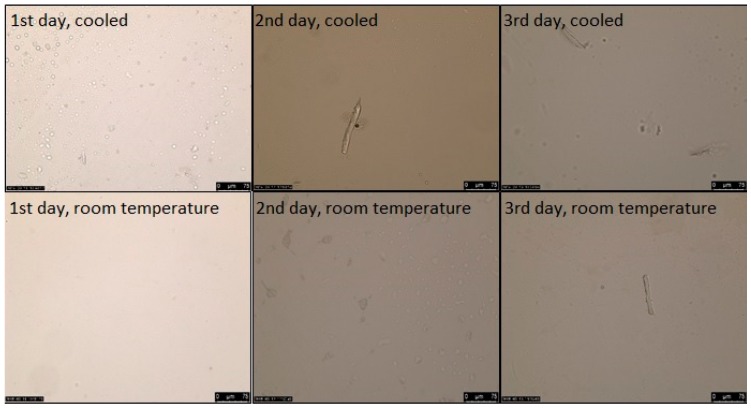
The NLC dispersions’ stability over the course of three days at different storage conditions. (The magnification was 200×).

Parallel to the particle size analysis, microscopic studies were performed as well on the NLC dispersions. As it can be seen in Figure 1, in cooled circumstances lidocaine crystals appeared on the second day, while at room temperature they appeared on the third day. Based on the microscopic examination, the holding time of the NLC dispersion was two days at room temperature.

**Figure 2:**
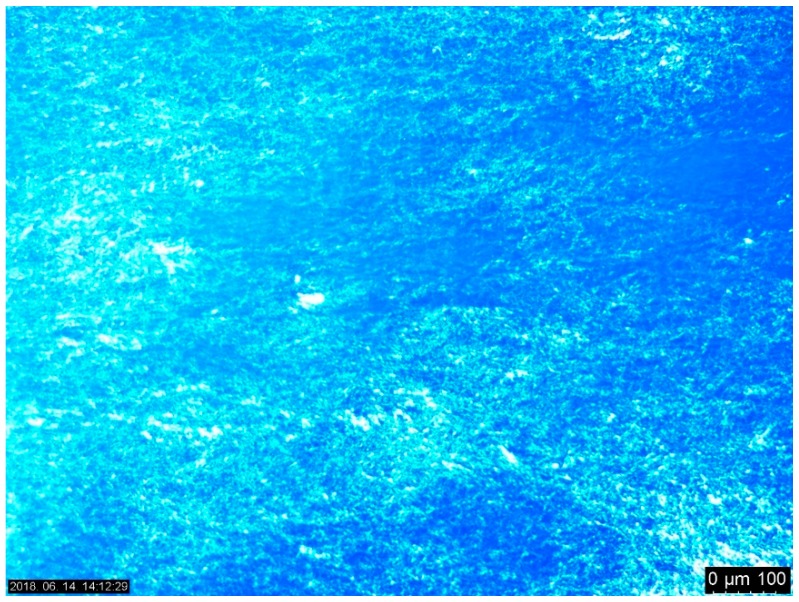
The solid lipid particles (LID-NLC) gel in polarized light at week two. (The magnification was 100×).

The structure of the LID-NLC gel was examined with a polarized light microscope as well. The formula had a homogenous structure after two weeks, no API crystals were detected (Figure 2). It can be concluded based on these results that it is very important to gelify the NLC dispersion as soon as possible for proper stability.

### 3.3. Rheological Studies

**Figure 3:**
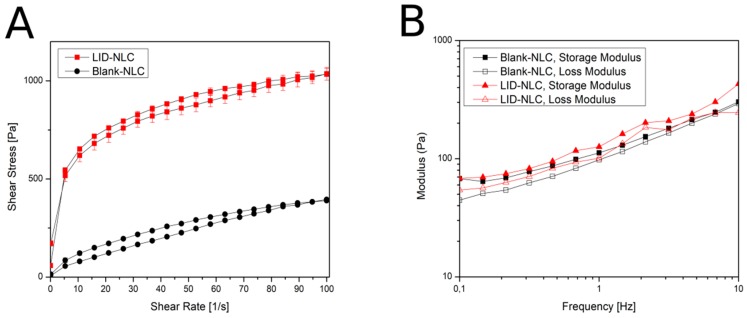
Rheological behavior of the NLC gel. (**A**): flow curves; (**B**): frequency sweep test.

The LID-NLC gel had a shear thinning behavior (Figure 3A), which means the shear stress increased continuously with the shear rate, but the rate of the increase decreased. Slight thixotropy was observed, meaning that structure regeneration was time-dependent. The viscosity value of the LID-NLC gel at the shear rate of 100 1/s was 6.72 Pa·s at 25 °C. According to the rheological measurements, the consistency of the formulation was suitable for dermal use. The viscosity value of the blank-NLC gel at the shear rate of 100 1/s was 5.58 Pa·s at 25 °C. Based on the rheological curves, it can be stated that incorporation of the API into the formulation made the system more viscous. Viscosity values increased and consistency improved. The frequency sweep test (Figure 3B) showed a viscoelastic behavior, where the elastic characteristic dominated (G′ higher than G″); with a relatively large frequency dependence of the moduli (G′ and G″) which is a typical feature of a so-called weak gel.

### 3.4. pH Measurement

pH of the blank-NLC dispersion was 5.2 ± 0.2 and the blank-NLC gel 5.3 ± 0.3. pH of the LID-NLC dispersion was 8.0 ± 0.3 and the LID-NLC gel 7.9 ± 0.4. Due to its basic characteristics, the active ingredient increased the pH value of the preparation.

### 3.5. Drug Release, Diffusion and Penetration Studies

The in vitro diffusion/penetration of lidocaine from the LID-NLC gel through the two synthetic and one biological membranes and the Skin-PAMPA membrane were calculated in terms of the mean cumulative amount and percentage diffused/penetrated ± SD by 6 or 24 h (Table 4).

**Figure 4:**
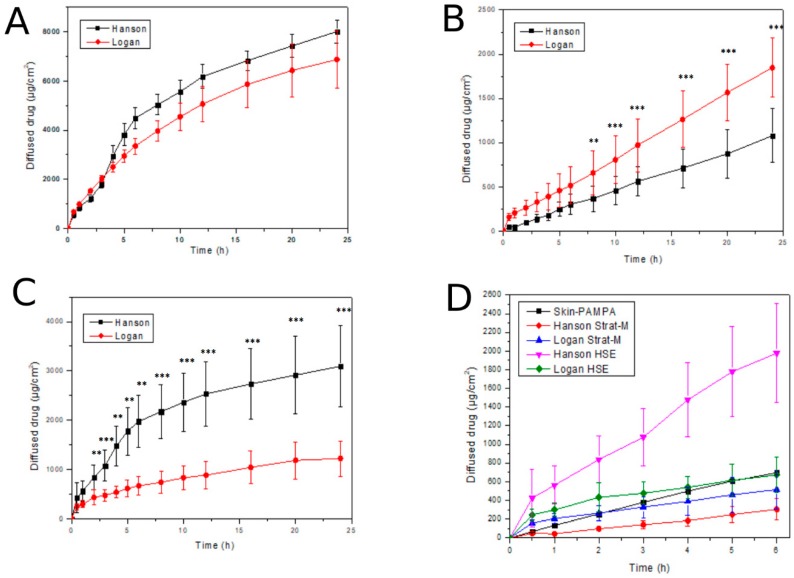
In vitro drug diffusion and ex vivo skin penetration studies. (**A**): In vitro release of lidocaine through cellulose acetate membrane (24 h); (**B**): In vitro penetration of lidocaine through Strat-M^®^ membrane (24 h); (**C**): Ex vivo penetration of lidocaine through HSE (24 h); (**D**): Comparison of various penetration methods with various membranes (6 h). *p* < 0.05 *, *p* < 0.01 ** and *p* < 0.001 *** compared to each other within each one figure.

The cellulose acetate membrane is a non-barrier, non-skin like membrane, which was used to measure the in vitro release of lidocaine from the NLC gel. As expected, a high amount of lidocaine released through this membrane over the course of 24 h from both Franz cell types (Figure 4A). Differences between the Logan and Hanson Franz cell results were not significant, however, a lower standard deviation was observed with the Hanson method.

The Strat-M^®^ membrane and HSE were skin-like and acted as barriers to API penetration. Figure 4B shows the penetration of lidocaine via the synthetic Strat-M^®^ membrane, which Figure 4C shows the penetration via HSE. As for the Strat-M^®^ membrane, differences between the Logan and Hanson Franz cells were significant after eight hours. Penetration via the HSE was higher than via the Strat-M^®^ membrane, especially on Hanson device over the 24 h. In the case of HSE, differences between the Logan and Hanson Franz cells were significant from two hours. This significant difference between the two types of Franz cells can be explained by the difference in structure of the two devices. The Logan cell was open from above, therefore the measurement runs on atmospheric pressure. However, the cell of the Hanson device was closed from the top and the pressure is higher leading to significantly higher penetration. The standard deviation was similar with the two methods. Figure 4D presents the diffusion/penetration data via the three different membranes (Strat-M^®^, Skin-PAMPA, HSE) tested on the Franz cells and Skin-PAMPA system. A lower standard deviation was observed with the PAMPA model than with the Franz cell method. This may be caused by the diversity in the membrane structure of the two methods. However, the penetration profiles of the Skin PAMPA and in vitro and ex vivo Franz cell methods were in good balance. If comparing the results at 6 h, the values obtained with Logan cells and Skin-PAMPA method were close to each other. Our results demonstrated that a novel Strat-M^®^ synthetic membrane and the Skin-PAMPA method have the potential to be used as an early screening tool to select the best dermal lidocaine-loaded nanoformulation.

## 4. Conclusions

A lidocaine-loaded NLC dispersion and a lidocaine-loaded, NLC gel were formulated to study their stability at various temperatures and evaluate their in vitro release as well as in vitro and ex vivo skin penetration. The NLC dispersions were found to be stable at room temperature, with acceptable changes in particle characteristics, for two days. Microscopic evaluations further verified the stability of the NLC dispersion at room temperature. It can be concluded that two days is a reasonable time period to create a gel from this NLC dispersion. The microscopic and rheological measurements verified that the consistency of the formulation was well suitable for dermal use, and the gel was stable for at least two weeks at room temperature.

Synthetic membranes are useful tools to examine and determine which formulation is the most promising for in vivo human skin studies. There are numerous commercial membranes available, all of which may have different drug diffusion properties. Cellulose membranes were used to study in vitro drug release, while the Skin-PAMPA membranes and Strat-M^®^ membranes were used for modeling in vitro skin penetration. Both the Skin-PAMPA and Strat-M^®^ membranes correlated well with HSE in this study, but the Strat-M^®^ membranes shared the most similar drug permeability profile to an ex vivo human skin model. Our results can be used to guide formulators in selecting vehicles in early development in the pharmaceutical, personal care and cosmetic industries. Overall, researchers must carefully select the most suitable membrane to be used with Franz cells for topical quality control testing.

Modeling of penetration through the skin is a complex challenge. The drug release profiles of the LID-NLC gel obtained with the different techniques were not fully equivalent. The device and the membrane, along with the properties of the product itself, influence how the system can be tested most effectively. Based on our results, we can select the best in vitro test for modeling human skin penetration of NLC gel systems, but appropriate in vitro/in vivo correlation should be performed to calculate the drug release in vivo. Future studies in this field are still needed to explain the influence of membranes and equipment from other classes on other drug candidates.

## Figures and Tables

**Table 1:** Compositions of the test preparations.

LID-NLC	Blank NLC	LID-NLC Gel	Blank NLC-Gel
Apifil 11.8%	Apifil 11.8%	LID-NLC 89%	Blank NLC 89%
Cremophor RH 60.8%	Cremophor RH 60.8%	Glycerin 8%	Glycerin 8%
Miglyol 812 N 5%	Miglyol 812 N 5%	Methocel E4M 3%	Methocel E4M 3%
Purified water 69.2%	Purified water 75.2%		
Lidocaine 6%			

**Table 2:** The experimental design of drug diffusion and penetration studies.

Device	Hanson	Logan	Skin-PAMPA
Membrane	Cellulose acetate	Cellulose acetate	Skin-PAMPA membrane
	Strat-M^®^	Strat-M^®^	
	HSE	HSE	

**Table 3:** Particle size of nanostructured lipid carrier (NLC) dispersions stored at different conditions.

Day	NLC Dispersion	d(0.1) µm	d(0.5) µm	d(0.9) µm	Span
1st day	Blank-NLC, room temperature	0.066	0.131	0.320	1.950
	Blank-NLC, cooled	0.066	0.130	0.302	1.821
	LID-NLC, room temperature	0.069	0.098	0.145	0.787
	LID-NLC, cooled	0.069	0.098	0.146	0.790
2nd day	Blank-NLC, room temperature	0.065	0.127	0.266	1.584
	Blank-NLC, cooled	0.065	0.127	0.266	1.587
	LID-NLC, room temperature	0.068	0.097	0.144	0.782
	LID-NLC, cooled	0.073	0.114	0.259	1.625
3rd day	Blank-NLC, room temperature	0.065	0.126	0.258	1.542
	Blank-NLC, cooled	0.065	0.126	0.262	1.564
	LID-NLC, room temperature	0.069	0.098	0.146	0.791
	LID-NLC, cooled	0.079	0.130	0.300	1.698
4th day	Blank-NLC, room temperature	0.065	0.128	0.289	1.746
	Blank-NLC, cooled	0.065	0.128	0.298	1.814
	LID-NLC, room temperature	0.090	0.163	41.749	255.201
	LID-NLC, cooled	-	-	-	-

**Table 4:** The mean cumulative amount and percentage (%) diffused or penetrated at 6 and 24 h (µg/cm^2^).

Time of Experiment	Cellulose		Strat-M^®^		HSE		Skin-PAMPA
	Hanson	Logan	Hanson	Logan	Hanson	Logan	
6 h (µg/cm^2^)	3808.82 ± 448.91	3355.40 ± 320.70	304.20 ± 113.40	514.89 ± 209.69	1778.25 ± 483.81	670.85 ± 189.05	696.32 ± 20.50
6 h (%)	41.35 ± 18.8	27.73 ± 0.83	3.45 ± 1.21	7.72 ± 2.89	11.61 ± 2.95	8.785 ± 2.03	13.93 ± 0.41
24 h (µg/cm^2^)	8014.05 ± 471.89	6878.13 ± 1172.21	1079.27 ± 304.36	1847.08 ± 335.19	3094.60 ± 829.73	1222.69 ± 358.70	-
24 h (%)	70.40 ± 13.72	58.66 ± 4.28	12.96 ± 3.52	27.89 ± 5.17	19.44 ± 5.05	16.00 ± 3.81	-
